# The Effect of Wood Chips with Various Degrees of Toasting on the Quality and Sensory Parameters of Fruit Distillates

**DOI:** 10.3390/molecules31152691

**Published:** 2026-08-02

**Authors:** Tomasz Tarko, Maryna Liubko

**Affiliations:** 1 Department of Fermentation Technology and Microbiology, University of Agriculture in Krakow, ul. Balicka 122, 30-149 Krakow, Poland; maryna.liubko@student.urk.edu.pl; 2 Center for Innovation and Research on Prohealthy and Safe Food, University of Agriculture in Krakow, ul. Balicka 104, 30-149 Krakow, Poland

**Keywords:** toasted wood, fruit distillates, aging, quality parameters, sensory parameters

## Abstract

The quality of fruit distillates is primarily influenced by how they are aged in wood. Not only the species of wood used but also the degree of its toasting is significant. The aim of this study was to analyze the effect of aging apple distillate using wood chips made from various tree species (oak, cherry, chestnut, juniper) with different degrees of toasting on its quality. The distillates were analyzed for total polyphenol content and antioxidant activity (spectrophotometric methods), acidity and ester content (titrimetric methods), color parameters (CIELab), and volatile compound profile (GC-MS). A sensory analysis of the distillates was also performed. The use of chestnut wood yielded the best results compared to other wood species, and the medium toasting level was the most suitable. Distillates made with oak and cherry wood produced similar, albeit slightly inferior, results, particularly with regard to the concentration of active compounds. However, high-temperature toasting of cherry wood chips did not positively affect the chemical composition of the distillates. Distillates made with juniper wood chips exhibited atypical physicochemical and sensory characteristics compared to those made from other wood species. In many cases, the distillates were similar to the control samples.

## 1. Introduction

Fruit distillates are popular beverages worldwide. They are produced from fruits with high sugar content, such as cherries, apples, grapes, and plums, and are appreciated for their unique flavor and aroma. The profile of volatile compounds, which includes components derived from the raw material as well as those formed during fermentation, distillation, and aging, plays a key role in shaping the beverages’ organoleptic characteristics and depends on the type of fruit, the quality of the raw material, and the production conditions [1]. The most popular fruit distillates are made from grapes, apples, plums, cherries, pears, and citrus fruits [2,3].

Fruit distillates vary in quality and sensory profile not only depending on the raw material used but also on the production process. The production of fruit distillates involves various stages, including mashing, fermentation, distillation, and aging. The parameters of each stage may vary, affecting the sensory properties [4]. During fermentation, the strain of yeast used is important. In addition to ethanol, yeast synthesizes various compounds, mainly acids, alcohols, esters, and carbonyl compounds, which directly or indirectly influence the aroma and flavor of the distillates [5,6]. During distillation, the main fraction—the so-called “heart”—is collected. This fraction contains most of the ethanol and volatile compounds responsible for the distillate’s characteristic aroma [7].

Aging is one of the most important stages that shapes the aroma and flavor of distillates. During this process, a series of slow chemical transformations take place, the most important of which are oxidation, esterification, and the partial breakdown of certain volatile compounds. As a result, the sharp, “raw” notes of the fresh distillate gradually mellow, and the aromatic profile becomes more harmonious and integrated. During aging, the proportion of esters responsible for pleasant fruity and floral notes increases as organic acids react with alcohols. At the same time, the concentration of certain aldehydes and higher alcohols, which can give the distillate a sharp, harsh character, decreases. The rate and extent of these changes depend on storage time, temperature, oxygen exposure, and the chemical composition of the distillate [8,9,10].

A very important aspect of aging is the distillate’s contact with wood while aging in barrels. The most commonly used woods for barrel production are oak (*Quercus*) and chestnut (*Castanea*). In recent years, there has been growing interest in using other wood species, such as cherry (*Prunus avium*), acacia (*Robinia pseudoacacia*), and mulberry (*Morus alba*) [11,12,13]. Distillates exposed to wood acquire complex aromatic profiles through the extraction of chemical compounds present in the wood. Among these components, ellagitannins, volatile phenols, lactones, aldehydes, and furan compounds can be distinguished; these are responsible for the characteristic vanilla, coconut, woody, and spicy aromas [11,14]. Ellagitannins are also responsible for the bitterness and astringency of beverages aged in wood [13,15].

Toasting wood is a commonly used process in cooperage and wood chips production. This process modifies the physical structure of the wood, resulting in a change in its chemical composition due to the thermal degradation of wood polymers. The transformation of volatile compounds depends heavily on temperature and exposure time [14,16]. The thermal degradation of hemicellulose produces numerous volatile compounds that are responsible for the characteristic aromas of toasted wood. These compounds include furfural, 5-methylfurfural, 5-hydroxymethylfurfural, and furfuryl alcohol. Concurrently, Maillard reactions occur, leading to the formation of cyclothene, maltol, furanol, and dihydromaltol, among others, which have a caramel-like and sweet odor [17,18,19]. The thermal decomposition of lignin produces volatile phenols, phenolic aldehydes, phenolic alcohols, phenyl ketones, and phenolic esters and acids. These compounds contribute to the characteristic aromas of toasted wood, including vanilla, spicy, and smoky notes [20,21].

Using wood chips instead of barrels to age distillates is becoming increasingly popular. It allows distillates to be aged in metal tanks, which facilitates temperature control and cleaning and reduces losses associated with the evaporation of distillate from barrels. In addition, adding wood chips accelerates the aging process compared to traditional barrels. It also enables the use of wood species that cannot be used to make barrels and is significantly cheaper than traditional aging methods [12,22,23].

This study was designed to utilize wood species used in traditional cooperage, as well as those not commonly used for aging alcoholic beverages. The first group includes oak and chestnut. In addition, it was decided to use cherry wood, which is characterized by high hardness compared to other fruit trees, and juniper wood chips. Juniper was chosen because there are no reports in the literature concerning wood chips from this shrub, and due to the rich composition of essential oils in its seeds. Most of the experiments on the aging of fruit distillates described in the literature focus on grape brandy. There are, however, few reports describing the aging of apple brandy.

The aim of this study was to analyze the effect of aging using wood chips from various wood species (oak, cherry, chestnut, and juniper) and their degree of toasting on the sensory profile of apple distillate. Two research hypotheses were formulated: (1) the use of wood chips from different tree species with varying degrees of toasting will improve the physicochemical parameters of apple distillates; (2) the use of cherry and juniper wood chips with varying degrees of toasting will improve the sensory parameters of apple distillates.

## 2. Results and Discussion

### 2.1. The Impact of Wood Chips with Varying Degrees of Toasting on the Antioxidant Activity and Total Polyphenol Content of Apple Distillates

Polyphenols and furan compounds are among the key wood-derived substances that migrate into distillates during aging. Their content depends on several factors, among which the wood species, its thermal treatment, and the duration of the aging process are of key importance [24].

**Figure 1:**
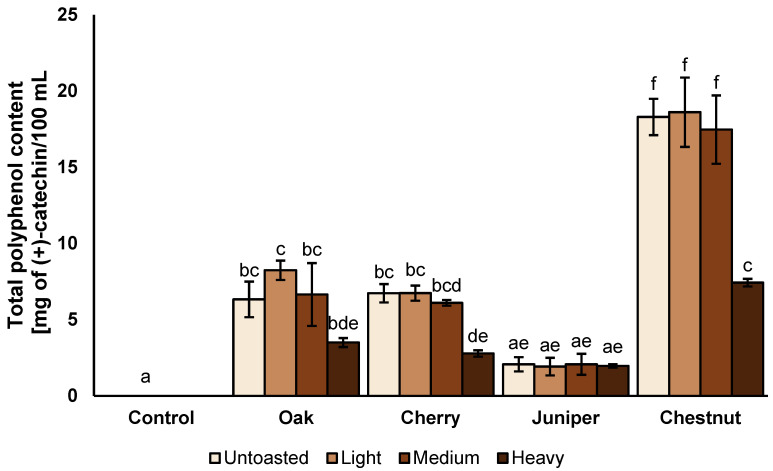
Total polyphenol content of apple distillates aged with wood chips from various tree species with different degrees of toasting. The same letters indicate no statistical significance at *p* < 0.05 (*n* = 3; mean ± SD).

**Figure 2:**
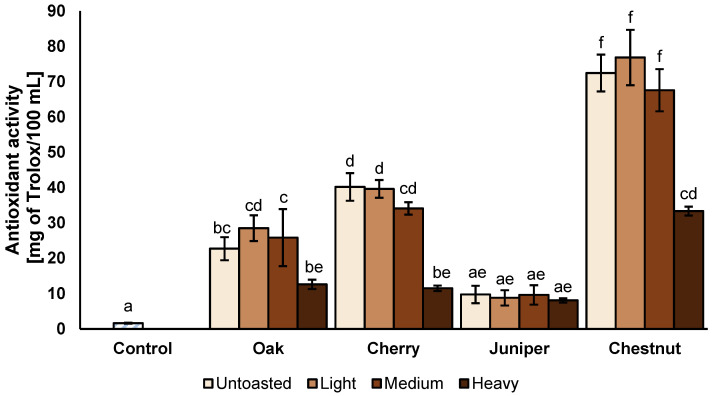
Antioxidant activity of apple distillates aged with wood chips from various tree species with different degrees of toasting. The same letters indicate no statistical significance at *p* < 0.05 (*n* = 3; mean ± SD).

Significant differences in total polyphenol content were observed depending on the wood species used (Figure 1). Among samples made from untoasted wood, the distillate with chestnut wood chips had the highest total polyphenol content, while the juniper sample had the lowest. The values obtained for oak and cherry wood were similar, and the differences between them were not statistically significant. A similar trend was observed for antioxidant activity, which was strongly correlated with total polyphenol content (R^2^ = 0.976). The distillate containing untoasted chestnut and cherry wood exhibited the highest antioxidant activity. Distillates made with untoasted oak and juniper wood exhibited lower antioxidant activity (Figure 2).

Similar trends in the phenolic compound content and antioxidant activity of the distillates were observed in studies by De Rosso et al. [25] and Híc et al. [26]. The authors demonstrated that samples aged using chestnut wood had the highest polyphenol levels. The high content of phenolic compounds and antioxidant activity of distillates aged with chestnut wood chips may result from the specific chemical properties of this wood. Chestnut wood contains low-molecular-weight phenolic compounds (e.g., gallic acid), tannins, and other active compounds that easily migrate into the distillate [25,27,28]. Cherry wood, despite its higher porosity, exhibits less variation in the levels of phenolic compounds, such as catechin and naringenin. Oak wood contains a broader profile of phenolic compounds; however, some of these compounds have a higher molecular weight and lower solubility, which may limit their extraction [29]. Juniper (*Juniperus* spp.) is a plant belonging to the Cupressaceae family and is classified as an evergreen shrub or small coniferous tree. It is characterized by a compact and simple anatomical structure, as well as high density and resistance to moisture [30]. The main constituents of juniper wood are essential oils, despite its relatively low polyphenol content. These oils are dominated by α-pinene, limonene, sabinene, and terpinen-4-ol, which exhibit some antioxidant activity [31,32].

Samples with lightly toasted wood had the highest antioxidant activity and total polyphenol content for distillates made from chestnut and oak wood. In contrast, increasing the toasting temperature and time resulted in a significant decrease in antioxidant activity. The lowest values were recorded for heavily toasted wood chips, possibly due to the degradation of more sensitive phenolic compounds or reduced wood porosity (Figure 2). The absence of statistically significant differences between untoasted, lightly toasted, and medium toasted samples suggests that medium toasting does not significantly affect polyphenol extraction. These findings are consistent with those of Cadahía et al. [33], who demonstrated that only intense toasting leads to a significant degradation of phenolic compounds. Another study [34] showed that toasting chestnut wood leads to a significant decrease in tannin content, especially at higher temperatures. A drastic decrease, mainly in antioxidant activity, but also in total polyphenol content, confirms that intense toasting not only leads to the breakdown of tannins but also reduces the wood’s potential to release phenolic compounds. This is probably due to structural changes (e.g., reduced porosity) or the thermal decomposition of sensitive compounds. Medium toasting contributes to the breakdown of high-molecular-weight lignins and leads to the formation of vanillin and syringaldehyde, which possess significant antioxidant potential. This also facilitates their extraction and increases the antioxidant potential of the distillates. Further toasting degrades labile antioxidants, leading to the formation of guaiacol and phenol according to the reaction described in [35]. These changes also result in a decrease in the antioxidant potential of apple distillates.

In contrast, the highest antioxidant activity and total polyphenol content were observed in distillates containing untoasted cherry wood chips. However, there was no statistical difference in the results obtained for the remaining samples.

The results obtained for the distillate with the addition of juniper wood chips differed from those obtained for distillates with tree species. The antioxidant activity and total polyphenol content did not change significantly with the degree of toasting. This may indicate the thermal stability of the essential oils contained in juniper. Unfortunately, juniper wood had not previously been studied in terms of toasting or its use as wood chips for aging vodka. Therefore, it is not possible to compare the results with those of other researchers. However, some authors have studied the behavior of various essential oils under the influence of high temperatures. In a study by Tomaino et al. [36], the authors demonstrated that essential oils are quite stable at high temperatures and that their antioxidant activity does not differ within the tested temperature range (80–180 °C). Only the content of α-pinene and sabinene decreased significantly after heating at 180 °C.

### 2.2. Effect of Wood Chips with Varying Degrees of Toasting on the Acidity and Ester Content in the Distillates

**Figure 3:**
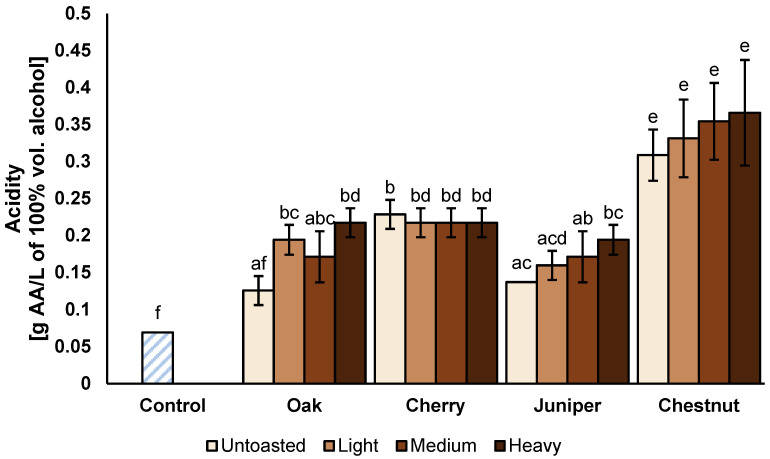
Acid content in apple distillates aged with wood chips from various tree species with different degrees of toasting. The same letters indicate no statistical significance at *p* < 0.05 (*n* = 3; mean ± SD).

The results for acid content in the tested distillates are presented in Figure 3.

The control sample had the lowest acidity (0.07 g/L), while the addition of wood chips significantly increased the acidity of the distillates (Figure 3). Among the distillates to which untoasted wood chips were added, those with chestnut wood showed the highest acidity (0.31 g/L). Distillates with cherry wood chips also exhibited high acidity (0.23 g/L). Samples aged with oak and juniper wood chips exhibited lower acidity (0.13 g/L and 0.14 g/L, respectively) and did not differ statistically. The increase in acidity observed in the distillates may result from the acidity of the wood used and the rate at which these compounds migrate into the distillates. Balaban and Ucar [37] demonstrated that chestnut wood had the highest acidity (91.2 mmol/100 g), while juniper had a significantly lower level (34.4 mmol/100 g).

Depending on the wood species, the degree of toasting affected the acidity of the distillates differently. For oak, an increase in the acidity of the distillates was observed as the intensity of toasting increased. However, the results for light, medium, and heavy toasting were not statistically different. This suggests that the toasting process facilitates the release of acidic compounds. In the experiments with juniper and chestnut, acidity increased with the degree of toasting. These trends can be linked to the thermal degradation of wood’s structural components, particularly hemicelluloses and lignin. When temperatures are very high, especially above 150 °C, the O-acetyl groups in hemicelluloses rapidly decompose, releasing significant amounts of acetic acid—the predominant organic acid formed during the heat treatment of wood. In the case of hardwood, approximately 40–50% of the O-acetyl groups decompose into acetic acid at temperatures of about 145–190 °C. This explains the significant increase in acidity of distillates containing hardwood with higher toasting levels [38]. Juniper wood, on the other hand, had a high lignin content. Although it contains a smaller amount of O-acetylated hemicelluloses, it can also generate acetic acid, primarily from the degradation of galactoglucomannan and lignin [39]. Furthermore, the thermal degradation of esters (Figure 3), following the use of higher toasting temperatures, may contribute to a slight increase in the acidity of distillates produced from wood chips. The release of the acidic character of esters, following the removal of the alcohol group, may to some extent increase the acidity of the distillates. It should be noted, however, that the differences in acidity were not statistically significant. In contrast, the opposite trend was observed for distillates made from cherry wood chips: the highest acidity occurred in the untoasted sample, and toasting resulted in decreased acidity. However, the differences were not statistically significant. It has been shown [40] that toasted cherry wood contains lower levels of lignin derivatives than oak wood, which may explain the results obtained in this study.

**Figure 4:**
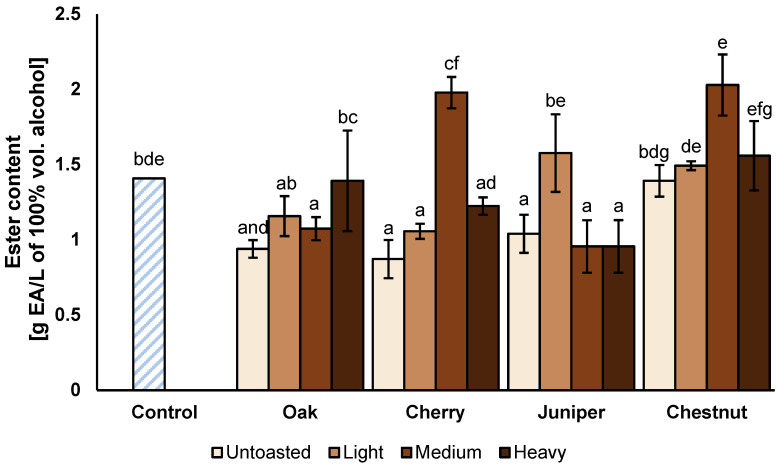
Ester content in apple distillates aged with wood chips from various tree species with different degrees of toasting. The same letters indicate no statistical significance at *p* < 0.05 (*n* = 3; mean ± SD).

The results of ester content in distillates are presented in Figure 4.

Significant differences in ester content can be observed among the tree species studied when comparing distillate samples with the addition of untoasted wood (Figure 4). Among the wood-aged samples, the distillate aged with chestnut wood chips had the highest ester content, while those aged with untoasted cherry, juniper, and oak wood had lower levels of these compounds. In comparison to the trials conducted with wood chips, the control trial exhibited the highest ester content. It has been shown [12] that the ethyl acetate content in control distillates and those aged with various species of untoasted wood are similar. However, the use of cherry wood exhibited different behavior; these wood chips caused a decrease in the amount of ethyl acetate in the distillates. In another study [41], the authors reported that during the aging process of alcoholic beverages, not only are compounds released into the beverage, but also aromatic compounds are absorbed from beverage into the wood. The absorption of esters by the wood was demonstrated, but to varying degrees. For example, ethyl octanoate was absorbed very well, while ethyl decanoate was not absorbed at all.

An analysis of the effect of wood toasting degree on the ester content in apple distillates shows that the intensity of heat treatment is significant, though this effect depends on wood species (Figure 4). For samples aged with chestnut wood chips, ester values increased from 1.39 g/L for untoasted wood to 2.03 g/L for medium toasted wood, while a further rise in the degree of toasting led to a reduction in their content in the distillates (1.56 g/L). A similar trend was observed for distillates with cherry wood added. The results obtained for distillates with juniper wood showed a different trend. Samples with lightly toasted wood chips had the highest ester content, and further thermal treatment decreased the ester concentration in the distillates (Figure 4). Terpenes, which are abundant in juniper wood chips, undergo decomposition (the breaking of carbon-carbon double bonds) at lower toasting temperatures, releasing alcohols. Alcohols may react with acids, increasing the concentration of esters in apple distillates. More intense heat treatment leads to the dehydrogenation of the rings, resulting in the formation of ketones and certain aromatic compounds, such as p-cymene [42]. Samples containing oak wood chips exhibited an increase in ester content with the degree of toasting, ranging from 0.94 g/L for untoasted wood to 1.39 g/L for heavily toasted wood. It has been demonstrated [41] that wood porosity increases when exposed to high temperatures, which was caused by the degradation of the wood’s structural components. Cherry wood underwent the most significant changes, which in the present study may have led to increased migration of compounds into the distillate and such a sharp increase in the ester content of the distillate (Figure 4). The BET (Brunauer–Emmett–Teller) surface area was not determined in this study. It should be noted, however, that the particle size of the wood chips for all wood species was similar. The changes in ester content in the distillates with the addition of different wood species varied and depended on the degree of thermal treatment. Therefore, changes in wood porosity, which depend on temperature and wood species, appear to be the most likely explanation for these variations. Unlike the chips from other wood species, the ester content of oak wood chips was closely correlated with acidity. When wood chips with a medium or strong degree of toasting were used in the remaining samples, a decrease in ester content and an increase in acid content were observed (Figure 3 and Figure 4). Sundqvist et al. [38] found that thermal treatment of wood causes the breakdown of acetate and formate esters, leading to the formation of acetic and formic acids, respectively.

### 2.3. Effect of Wood Chips with Varying Degrees of Toasting on the Color Parameters of Distillates

An analysis of apple distillates aged with different types of wood of various degrees of toasting revealed that both the type of wood and the intensity of its thermal treatment clearly influence the color of the final product (Table 1). Among samples with untoasted wood, the highest lightness (L* = 94.82) was observed in distillates with added juniper wood chips, indicating an extremely light, nearly transparent color. The color had a slightly yellowish hue (b* = 3.78) and very low saturation (C*_ab_ = 3.79), while the value of a* (−0.35) indicated a shift toward a greenish hue. The control sample yielded similar color results (L* = 95.36), but the colorless appearance (b* = 0.57) and low saturation (C*ab = 0.58) were similar to water.

Different results were obtained for distillates made with untoasted cherry wood. Despite their relatively low lightness (L* = 93.12), the distillates were characterized by an intense yellow hue (b* = 12.19) and high saturation (C*_ab_ = 12.21). Visually, this translates to a light golden or straw-colored hue. Importantly, with a light degree of toasting, the yellow hue deepened (b* = 15.99). With medium and strong toasting, a decrease in the b* parameter and saturation was observed, resulting in a distinctly yellow-amber color. With strong toasting, the a* value shifted in the negative direction (a* = −1.61), and the sample exhibited a greenish hue. The study by Híc et al. [26] yielded different results. Samples with the addition of cherry wood chips exhibited higher lightness compared to the results obtained in this study. As the degree of toasting increased, the lightness of the distillate decreased, though it remained high.

Distillates aged with chestnut wood exhibited the greatest variability and the strongest influence on color during heavy toasting. Lightness decreased significantly (L* = 90.91), while b* and C*_ab_ increased to 20.01 and 20.09, respectively, indicating a very deep, saturated color. For samples with untoasted chips of this wood, the values were significantly lower (Table 1), corresponding to a light, slightly straw-colored hue. The marked decrease in lightness and the concurrent increase in color saturation with each successive level of toasting intensity suggest that wood thermal decomposition products (including Maillard reactions and caramelization) strongly influence the migration of color compounds into the distillate.

As with chestnut chips, a decrease in the lightness of the distillates was observed with oak chips depending on the degree of toasting, accompanied by an increase in the yellow color component and saturation (Table 1). The samples acquired a classic golden color typical of aged oak distillates. The a* values remained in the negative range, indicating the absence of a red hue and a predominance of green.

A study by Karathanos et al. [43] showed that distillates with the addition of chestnut had the highest a* and b* values and were more reddish-yellow in color. Furthermore, the distillate with chestnut wood was noticeably darker than the one with oak wood. These results differ from those obtained in the present study, in which distillates with chestnut and oak chips addition had lower b* values (though still in the positive range), while all samples had a* values in the negative range (approaching green). The lightness parameters were similar when using chestnut and oak wood chips. These discrepancies may be due to differences in aging time (the authors aged the distillate for five years) and the ratio of wood chip dosage to distillate volume. In all samples, the negative a* value differs from the values observed in barrel-aged distillates. In barrel-aged distillates, the a* value was usually positive, whilst the distillate, as a result of the extraction of various phenolic compounds, had a yellow-red color [44]. Such changes and deviations in the case of wood chips may be caused by the extraction of larger quantities of coumarin derivatives, as well as hydroxycinnamic and hydroxybenzoic acids, which have slightly different color properties to phenolic compounds [45,46]. Aging time is also an important factor influencing the color hue. In the early stages of aging, the distillates have a greenish hue, with minimal yellow tones. However, the longer the samples are aged in wood, the more red-colored compounds are transferred to the distillates [47]. Regardless of the degree of toasting, distillates with juniper chips had a very light and unsaturated color, with low b* values and relatively high lightness (Table 1). The distillate produced with these chips can be described as slightly yellowish and clear, not very different from the control samples. With intense toasting, a sharp increase in the b* and C*_ab_ parameters was observed.

In most cases, the hue values (h_ab_) fell within the range of 168–179°, confirming the dominance of yellow hues that transitioned to light gold in some cases. The highest h_ab_ values were observed in distillate samples with the addition of cherry wood chips at a medium toasting level, indicating a shift toward warmer, more orange tones. In contrast, lower h_ab_ values were observed for distillates aged with chestnut and oak wood, which exhibited deeper golden-brown tones (Table 1). The color parameters of distillates aged with oak and chestnut wood chips depend both on the degree of toasting and the wood species [48]. In particular, a high degree of barrel toasting leads to an increase in the h_ab_ value (yellow-red tones) and a decrease in lightness, which is associated with a higher polyphenol concentration. The a* values for lightly and heavily toasted barrels were −2.43 and 7.98 respectively. The L* lightness values, on the other hand, ranged from 92.76 (for lightly toasted barrels) to 80.33 (for heavily toasted barrels). More pronounced color changes were observed in distillates that had been in contact with chestnut wood than in those made from various oak species. The L* lightness value for distillates from oak barrels was 92.89, and for those from chestnut barrels it was 81.15. The a* color value was significantly higher for chestnut barrels (6.53) than for oak barrels (−1.33) [48]. In contrast, cherry wood had a smaller effect on the h_ab_ parameter, consistent with other studies [49], indicating a milder impact on the distillate’s color and allowing for the preservation of a more natural product color. The authors cited [49] demonstrated that samples aged with cherry wood chips were up to 20 per cent lighter in color than those aged with oak wood chips, particularly French oak. Unlike the previously described study, this work found that distillates made with cherry wood chips showed the greatest changes in the h_ab_ parameter. This may be related to the presence of a greater variety of flavonoids in cherry wood [50].

### 2.4. Effect of Wood Chips with Varying Degrees of Toasting on the Volatile Compounds of Apple Distillates

**Figure 5:**
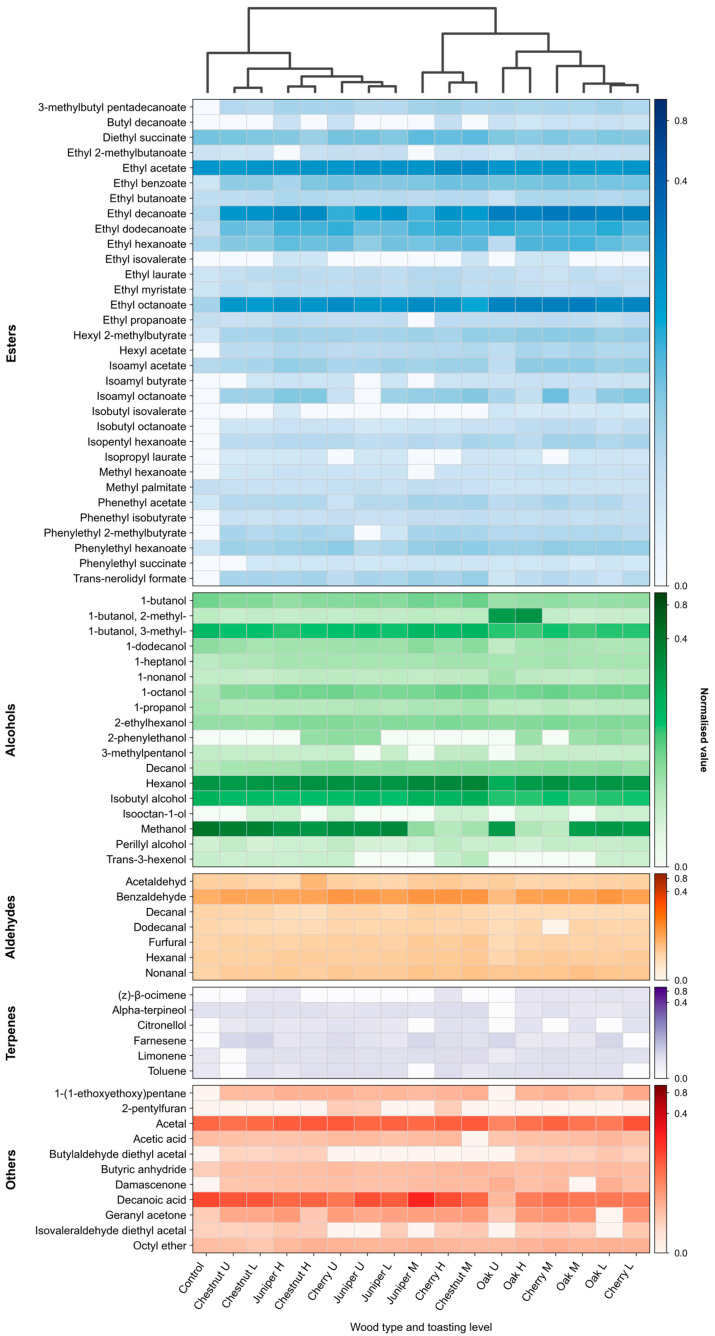
Hierarchical Cluster Analysis of volatile compounds in apple distillates (U—untoasted, L—lightly toasted, M—medium toasted, H—heavy toasted).

Chromatographic analysis of the distillate samples revealed the presence of 74 volatile compounds, which were classified into five groups: esters, alcohols, aldehydes, terpenes, and others. Hierarchical Cluster Analysis (HCA) in the form of a heat map (Figure 5) was used to visualize the results, considering the influence of wood species and the degree of toasting on the distillates. The HCA results are presented as a binary tree, or dendrogram. Individual samples were assigned to clusters based on similarity. Successive clusters are merged to form a dendrogram. The higher the level at which samples are merged, the more different they are from one another [51].

The cluster analysis revealed no specific groupings based on wood type or degree of toasting, suggesting that both parameters influence the distillates’ volatile compound profile. However, two main clusters with distinct compositional characteristics were identified. The first group included samples aged with untoasted, lightly toasted, and heavily toasted wood chips. These samples were similar to the control sample. The second group included the remaining samples, primarily those to which wood chips with a medium degree of toasting were added. A clear distinction between the compounds characteristic of specific groups in the dendrogram is not unambiguous. However, it may indicate the compounds typical of each group, as well as smaller proportions of other volatile components. Group one was characterized by the presence of decanoic acid, methanol and acetal. Simultaneously, smaller amounts of butyl decanoate, isobutyl isovalerate, 2-phenylethanol and β-ocimene were also detected. The second group of the dendrogram indicates consistently higher levels of ethyl dodecanoate, hexyl 2-methylbutyrate, isopentyl hexanoate and nonanal. However, a lower proportion of trans-3-hexenol was observed (Figure 5).

Significant differences were observed in the volatile compound profiles of the selected distillates. The control sample contained lower levels of esters, terpenes, and certain alcohols, and lacked ethyl amyl acetal, 2-pentylfuran, and damascenone. The most characteristic and most significant compounds that influence the sensory profile of raw distillates are ethyl acetate, 1-butanol, isoamyl alcohol, 2-methyl-1-butanol, 1-propanol, acetaldehyde, and furfural [12]. In the control sample, volatile alcohols were predominant. Their amount was more than 77% of the other compounds that were found. Higher alcohols impart spicy and slightly fruity aromas to the distillates [52,53]. The control sample contained a high concentration of hexanol (Figure 5). Hexanol, along with hexyl 2-methylbutanoate, is associated with green and apple-like aromas, and these compounds are characteristic compounds of apple distillates [3].

The control sample contained higher amounts of higher alcohols than the distillates aged with wood chips (Figure 5). For light and medium toasting levels, the differences were not significant, but adding heavily toasted wood chips to distillates decreased the content of higher alcohols markedly. Similar results regarding the reduction in higher alcohols have been reported by other authors [54,55,56]. This phenomenon may be related to the esterification process and the formation of new fatty acid esters, as well as the ability of heavily toasted wood to absorb higher alcohols [57].

The total ester content in the samples aged with wood chips increased significantly compared to the control samples. All samples aged with wood chips exhibited high levels of ethyl decanoate, ethyl octanoate, ethyl hexanoate, and ethyl dodecanoate (Figure 5). A similar effect was observed in the study by Arduini and Chinnici [55], in which the total content of ethyl esters in Italian brandy increased significantly after 8 months of aging compared to the control sample, mainly due to the accumulation of ethyl esters of medium-chain fatty acids (e.g., ethyl hexanoate, ethyl octanoate, and ethyl decanoate). However, the relationship with the degree of chips toasting described in that publication differed from that observed in the present study. The authors demonstrated that the ester concentration was higher in brandy made with lightly toasted wood chips than in brandy made with heavily toasted wood chips. In this study, the content of most esters remained similar or increased insignificantly as the degree of wood toasting increased. This increase in esters occurred through the esterification reaction of fatty acids and alcohols during the distillate aging [58].

Distillates made with oak wood chips had the highest levels of identified esters compared to those made with other wood species (Figure 5). Similar results were obtained in studies conducted by Duan et al. [22]; distillates aged with various wood species had higher levels of esters with a floral-fruity profile. Oak wood is characterized by higher levels of ethyl decanoate, ethyl octanoate, and ethyl 2-methylbutanoate compared to other wood species [22]. The HCA analysis classified all distillates with the addition of oak chips into the second group, indicating similarity in their chemical compositions. Distillates made with heavily toasted oak wood and untoasted oak wood were classified into a single statistical group. The similarity between wood with different degrees of toasting can be explained by the mechanisms of thermal degradation and oxidation of chemical compounds. During the toasting process, the wood’s structural components undergo gradual thermal degradation, leading to the formation of new volatile and aromatic compounds. As the temperature rises, the concentration of these compounds initially increases, reaching a maximum value characteristic of a given compound. Further exposure to high temperatures causes the compounds to decompose or oxidize, resulting in decreased concentration in the wood and, consequently, in the distillate [59]. The thermal treatment of oak wood resulted in the formation of a variety of compounds, including ethyl isovalerate, 2-phenylethanol, 3-methylpentanol, isooctanol, beta-ocimene, alpha-terpineol, citronellol, ethyl-amyl acetal, 2-pentylfuran, diethyl acetal of butyl aldehyde, and diethyl acetal of isovaleraldehyde. The opposite effect was observed for isoamyl octanoate and methanol; these compounds were more abundant in distillates from untoasted wood chips than in those from heavily toasted wood chips.

The distillates made from cherry wood chips were divided into two groups. The first group consisted of samples made from untoasted chips, and the second group consisted of the remaining samples. The distillates made with cherry wood do not cluster together, indicating significant differences between samples containing chips with varying degrees of toasting. Distillates made with untoasted cherry wood chips contained higher amounts of methanol, 2-phenylethanol, and 2-pentylfuran compared to the other samples. A very similar ester profile was observed in distillates made with untoasted cherry and oak wood chips (Figure 5). Other studies [59] have found that distillates made with cherry wood contain higher proportions of ethyl decanoate, ethyl propionate, and isobutyl acetate than samples made with oak wood. In the present study, these esters were identified at similar levels for both wood species (Figure 5). These compounds impart floral, fruity, and sweet aromas to the distillates, thereby accentuating the natural aroma of the raw material [59]. As the toasting level increased to light and medium, an increase in isoamyl octanoate and diethyl acetal of butyl aldehyde was observed, followed by a decrease in the concentration of these compounds. Distillates made with cherry wood contained high concentrations of decanoic acid (heavy toasted wood chips) and ethyl isovalerate (medium toasted) (Figure 5). Such differences in the composition of volatile compounds between samples aged with wood of varying degrees of toasting may be due to the wood’s porosity and the intensity of compound extraction. Cherry wood is quite porous, allowing for greater extraction of compounds, even from untoasted wood chips, compared to other wood species [24,50]. High temperatures and changes in the wood’s structure increase the porosity of cherry wood. Intense toasting of the wood chips also causes cracks in the wood’s structure, which facilitates penetration by the distillate and accelerates extraction [12,22]. As the pyrolysis temperature of wood increases, compounds such as furans, aldehydes and ketones, as well as acids and esters, are formed [60], which may also explain the increased proportion of acids and esters in medium- and heavily toasted cherry wood chips. At higher temperatures (heavy toasting), ester bonds break down, and decarboxylation removes the carboxyl group from phenolic acids, leading to the formation of alcohols [61].

The samples of distillates containing juniper chips are quite clustered closely together on the dendrogram. Distillates made with untoasted juniper, as well as those made with lightly and heavily toasted juniper wood, were classified into a single, larger group (Figure 5). The toasting process resulted in the formation of compounds not found in the sample with untoasted wood, such as isobutyl octanoate and phenethyl 2-methylbutyrate. Some compounds (2-phenylethanol and 2-pentylfuran) degraded due to thermal treatment. An interesting result was observed in distillates made with heavily toasted juniper: high temperatures led to the formation of ethyl isovalerate, butyl decanoate, isobutyl isovalerate, trans-3-hexenol, isooctanol, (z)-beta-ocimene, and diethyl acetal of butyl aldehyde. Distillates from medium toasted juniper were placed in a separate group (Figure 5). These distillates did not contain ethyl propionate, methyl hexanoate, isopropyl laurate, or citronellol. They also had a lower methanol content compared to the other samples. Juniper’s main components are monoterpenes, oxidized monoterpenes, and sesquiterpenoids, and their proportions vary depending on the species of shrub [62,63,64]. The dominant compounds in juniper wood are limonene (19%), α-terpinyl acetate (9.1%), β-phellandrene (8.9%), α-terpineol (8.4%), and α-pinene (7.5%) [63]. Contrary to expectations, juniper wood chips did not introduce a significant amount of terpenes. Compared to the control sample and samples from other wood species, the juniper samples did not differ significantly in terms of terpene composition. The terpenoid profile was similar to that observed for samples containing cherry wood (Figure 5). The differences in the volatile compounds profile between the control sample and the samples containing juniper wood chips in this study were mainly due to changes in the esters and higher alcohols fractions, rather than the terpene fraction. The extraction of terpenes depends on a number of factors: the type of terpenes, the polarity of the solvent, and the duration and temperature of the process. Pure terpenes are highly non-polar and practically insoluble in water, but become highly soluble as the concentration of ethanol increases. Oxidized terpenes are more soluble in water than terpene hydrocarbons due to the presence of hydroxyl groups. Pure ethanol extracts non-polar terpenes well, but the addition of water alters its polarity, allowing for the selective isolation of heavier sesquiterpenes and diterpenes. For example, the partition coefficient (logP) for limonene is 4.57, for α-pinene 4.83, but for menthol 3.40. Another important aspect is the temperature-dependent kinetics of diffusion. It has been shown that raising the temperature from room temperature to 40 °C results in a nearly twofold increase in the recovery of terpenes from herbs. Even better results can be achieved at 80 °C, but the volatility of terpenoids must also be taken into account [65,66,67].

Even though they were present in samples from other wood species, compounds such as butyl decanoate, isobutyl isovalerate, and 2-pentylfuran could not be identified in the distillates containing chestnut wood chips (Figure 5). Chestnut wood has a different chemical composition than other wood species. It contains high concentrations of ellagitannins, primarily gallic acid, vescalagin, and castalagin, as well as gallotannins not found in oak [40]. Distillates from untreated wood did not contain isoamyl butyrate, phenethyl succinate, limonene, or toluene, compared to the heat-treated samples (Figure 5). The absence of these compounds confirms that raw wood contributes significantly less aroma to alcoholic beverages than heat-treated wood [68].

### 2.5. Effect of Wood Chips with Varying Degrees of Toasting on the Sensory Parameters of Distillates

The results of the sensory scoring of the distillates are presented in Table 2.

The distillate made with heavily toasted chestnut wood chips received the highest rating of 4.11 points. This value was more than 30 per cent higher than the score obtained for the control sample. The distillate made with medium-toasted chestnut wood chips also received a high rating (3.93 pts.), which may indicate the wood’s great potential to enrich distillates’ flavor and aroma profile. Aged distillates with cherry wood chips received fairly high ratings as well. The highest ratings were observed when medium toasted wood chips were used (3.83 pts). This value was 23 per cent higher than that obtained for the control sample. Interestingly, these samples showed the least variation—regardless of the degree of toasting, they all scored between 3.71 and 3.83 points. In the case of oak wood, the highest score (3.83 points) was given to the distillate with the addition of medium-toasted chips. This value was identical to that obtained when using medium-toasted cherry wood chips. Heavy toasting, however, resulted in decreased sensory acceptance (3.21 points). Therefore, it can be concluded that excessive toasting intensity may negatively affect the taste and aroma of distillates with oak chips. Distillates with juniper wood received the lowest scores (3.02 and 3.08 for lightly and medium toasted wood, respectively). These values were 3 per cent and 1 per cent lower, respectively, than those obtained for the control sample. The sample with the addition of heavily toasted juniper wood chips received the higher score (Table 2), though it was still lower than that of distillates aged with heavily toasted cherry and chestnut wood. Notably, the control sample received nearly the lowest score (3.11 points), compared to the samples with added wood. This indicates a positive effect of aging with added wood on the sensory characteristics of the distillate. Only the distillates aged with juniper wood chips failed to meet the sensory panel’s approval.

**Figure 6:**
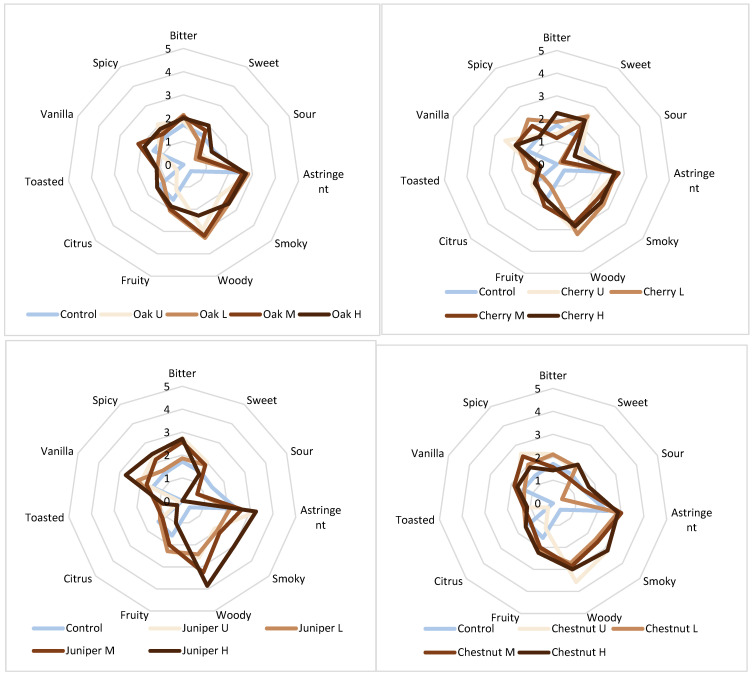
Effect of wood chip addition on the flavor profile of apple distillates (U—untoasted; L—lightly toasted; M—medium toasted; H—heavy toasted).

A more detailed evaluation of the flavor profile (Figure 6) revealed that nearly all samples aged with wood chips exhibited an intense woody and astringent flavor. For samples using oak wood, a strong degree of toasting reduced the perception of the woody flavor while increasing the intensity of the vanilla flavor. A similar trend was reported in the study by Chira and Teissedre [69]. An increase in the intensity of vanilla notes was observed for medium and heavy toasting levels, which was caused by increased vanillin extraction and the presence of lactones that enhance these flavor attributes.

As the degree of toasting increased, so did the perceptibility of the toasty flavor. This trend was observed for all types of wood (Figure 6), although in the case of chestnut wood, the intensity of this flavor remained unchanged.

Quite interesting results were observed with juniper wood. All samples exhibited woody, vanilla, bitter, and astringent flavors, and heavily toasted juniper produced the highest intensity of woody flavor among all wood types. However, in these trials, the sensory panel did not detect any fruity or citrus flavors. The intensity of these flavors decreased as the degree of toasting increased, indicating the degradation of fruity compounds. It has been shown [36,70] that essential oils responsible for pine, woody, and citrus notes (e.g., α-pinene and sabinene) degrade at temperatures above 180 °C.

An interesting result was observed in distillates made with lightly toasted cherry wood chips. These distillates had the highest intensity of toasty, spicy, and woody flavors and lacked fruity or citrus notes. In contrast, distillates made with untoasted wood exhibited an intense vanilla flavor. Sanz et al. [40] found that the degree of toasting affects the content of volatile compounds in cherry wood. They also demonstrated that untoasted cherry wood contains higher amounts of condensed tannins, vanillin, and a large amount of flavonoids, which are responsible for vanilla, bitter, astringent, and fruity notes. However, as the temperature increases during toasting, these compounds degrade. Additionally, the proportion of lignin degradation products, such as aromatic aldehydes (e.g., vanillin, protocatechuic aldehyde) and phenolic acids (e.g., benzoic acid, cinnamic acid), increases. These compounds are responsible for toasty, spicy, and woody notes.

Among the distillates made with chestnut wood, those made with untoasted wood chips exhibited the most intense woody and spicy flavors. However, these distillates contained the fewest fruity and citrus notes. In contrast, samples made with heavily toasted chestnut wood exhibited sweeter and less spicy notes. Furthermore, all types of wood (except juniper) showed an increase in the intensity of the sweet flavor as the degree of toasting increased. This is related to the degradation of hemicelluloses into simple sugars under the influence of temperature and their subsequent decomposition into compounds such as furfural, 5-methylfurfural, 5-hydroxymethylfurfural, and Maillard reaction products [17,18].

**Figure 7:**
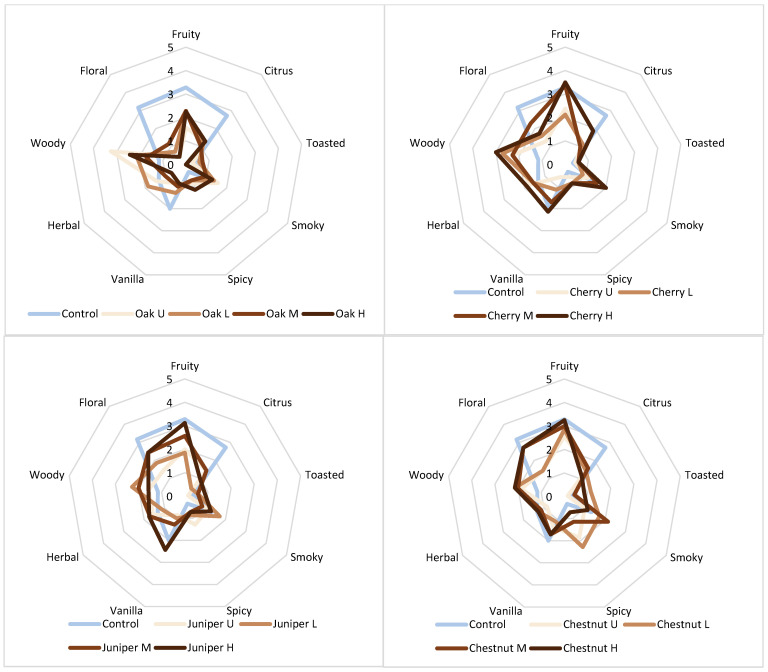
Effect of wood chip addition on the aroma profile of apple distillates (U—untoasted; L—lightly toasted; M—medium toasted; H—heavily toasted).

The distillates were also evaluated for the perceptibility of selected aromas, and the results obtained are presented in Figure 7.

The control sample exhibited fruity, floral, citrus, and slightly vanilla-like aromas. This is because apple distillate contains esters, which are produced during mash fermentation and are transferred partially to the distillates during distillation [22,71]. Among the compounds identified following chromatographic analysis (Figure 5), it can be seen that the fruity aroma of the control sample is likely attributable mainly to ethyl esters (e.g., diethyl succinate) and alcohols (e.g., 1-butanol or isobutanol). Floral notes are contributed, for example, by dodecanol or terpineol [72].

Samples made with untoasted oak had a fairly intense woody and smoky aroma, similar to samples with heavily toasted wood. The sensory panel also reported similar olfactory impressions in the other distillates containing oak chips. In this respect, the distillates containing oak chips were relatively consistent. The woody notes may be attributable (among those identified—Figure 5) to isobutyl isovalerate, nerolidyl formate and the terpenoids β-ocimene and farnesene. These compounds exhibit woody and fruity odors. Interestingly, heat-treated wood chips exhibited more intense fruity notes than untoasted wood chips (Figure 4). Hexyl alcohol esters and acetals (identified in this study—Figure 5) may be responsible for this. The peak areas for these compounds in samples containing untoasted oak were smaller than those in samples containing toasted wood chips.

Similarly, distillates with the addition of heavily toasted cherry wood chips exhibited high intensity of smoky, vanilla, and woody aromas, as well as strong fruity aroma. In this case, the intense woody aroma may be attributable to terpenes (β-ocimene and farnesene), which contribute such fragrance notes; the surface response for these was greater than for the samples containing untoasted and lightly toasted wood chips (Figure 5). In contrast, distillates aged with medium toasted cherry wood exhibited a floral aroma unlike other variants of this species of wood (Figure 7). Higher levels of, for example, ethyl dodecanoate with floral notes were found in these samples (Figure 5).

Distillates aged with heavily toasted juniper chips exhibited fruity and vanilla notes, while those aged with lightly toasted chips had more woody and smoky aromas (Figure 7). Furthermore, panelists detected the lowest intensity of citrus aroma in the juniper wood samples compared to other wood species. This result is surprising because the main volatile compounds in juniper are essential oils with predominantly citrus aromas. Barnes et al. [59] demonstrated that essential oils derived from juniper berries may contain compounds with woody and spicy notes, such as α-pinene, β-pinene, β-myrcene, and β-caryophyllene. Although juniper berries contain more terpenoids than the wood, their profiles in the wood and berries are similar [73]. However, the results obtained were corroborated by chromatographic analysis (Figure 5). None of the distillates from juniper wood chips stood out in terms of their content of compounds with a citrus aroma (e.g., limonene, terpineol, citronellol).

Samples containing heavily toasted chestnut wood chips were distinguished by the most intense fruity, floral, and woody notes. Substances such as ethyl decanoate, ethyl octanoate and phenethyl acetate may be responsible for such olfactory sensations (Figure 5). In contrast, distillates made with lightly toasted chestnut chips exhibited the most intense spicy aroma (Figure 7). This aroma may be associated with geranyl acetone (Figure 5). The intense toasty aromas, on the other hand, may be due to a relatively high proportion of furfural.

## 3. Materials and Methods

### 3.1. Materials and Chemicals

The apple wines were produced using fruit from the Red Boskoop and Koksa Pomaranczowa varieties (the pomological orchard of the University of Agriculture in Krakow, Poland). The wood chips used in the study were derived from various species of wood. The chestnut, American oak, and cherry wood chips were provided by “Arôbois” (Gagnac-sur-Cère, France), and the juniper wood chips were supplied by “Malinowy Nos” (Katowice, Poland). All the wood chips were of similar dimensions (5–8 mm) and were toasted under the same temperature conditions (SML-32 dryer, Zalmed, Warsaw, Poland). Toasting parameters: light (110 °C, 90 min); medium (170 °C, 120 min); strong (220 °C, 170 min). The full experimental design covered all possible combinations of wood type and degree of toasting. The control sample consisted of distillate without the addition of wood chips.

The diammonium salt of 2,2′-azino-bis(3-ethylbenzothiazoline-6-sulfonic) acid (ABTS diammonium salt), (±)-6-hydroxy-2,5,7,8-tetramethylchromane-2-carboxylic acid (Trolox), and phosphate-buffered saline (PBS: 0.01 M phosphate buffer, 0.0027 M potassium chloride, 0.137 M sodium chloride; pH 7.4 at 25 °C) were obtained from Sigma-Aldrich (Saint Louis, MO, USA). Sodium hydroxide (NaOH), hydrochloric acid (HCl), sodium carbonate (Na_2_CO_3_), sodium pyrosulfite (Na_2_S_2_O_5_), and Folin-Ciocâlteu reagent were obtained from POCh (Gliwice, Poland).

### 3.2. Wine Preparation and Aging of Distillates

The apple wine was produced from a 1:1 blend of the Red Boskoop and Koksa pomaranczowa varieties. The wine was then subjected to straight distillation, yielding a distillate with an alcohol content of 45% (*v*/*v*). The distillate was then subjected to fractional distillation, from which a middle fraction of 57% vol. was collected and diluted to 45% vol. Subsequently, 0.25 g of wood chips from a specific tree species were added to 100 mL of this distillate and aged for 30 weeks at a temperature of 20 °C. The experiments were carried out in our university’s aging chamber. The room was designed for the aging of alcoholic beverages under controlled conditions, away from light. For the purposes of these analyses, a temperature of 20 °C was used.

All tests and analyses were conducted in at least three physical replicates.

### 3.3. Antioxidant Activity (AOX)

Antioxidant activity (AOX) was determined according to the methodology described by Re et al. [74], using the active cation radical 2,2′-azino-bis(3-ethylbenzothiazoline-6-sulfonic) acid (ABTS, Sigma-Aldrich; Saint Louis, MO, USA). AOX was calculated based on a calibration curve prepared each time with synthetic vitamin E (Trolox, (±)-6-hydroxy-2,5,7,8-tetramethylchromane-2-carboxylic acid, Sigma-Aldrich; Saint Louis, MO, USA) and expressed as mg of Trolox per 100 mL.

### 3.4. Total Polyphenol Content (TPC)

The total polyphenol content (TPC) was determined using the Folin-Ciocâlteu method, as previously described in detail by Tarko et al. [75]. The results were expressed as mg of (+)-catechin per 100 mL.

### 3.5. Determination of Acidity *[76]*

50 mL of distilled water was added to 50 mL of distillate. The mixture was heated in a water bath under a reflux condenser for 30 min. Subsequently, the sample was cooled and titrated with 0.1 M NaOH using phenolphthalein as an indicator. The acidity was calculated based on the amount of reagent used and expressed as grams of acetic acid per liter of 100% distillate.

### 3.6. Determination of Ester Content *[77]*

After determining the acidity, 10 mL of 0.1 M NaOH was added to the neutralized sample and the mixture was heated under a reflux condenser for one hour. Then, the sample was cooled and 10 mL of 0.1 M HCl was added. The mixture was then titrated with 0.1 M NaOH against phenolphthalein. The amount of reagent used was utilized to determine the ester content, which was then expressed as grams of ethyl acetate per liter of 100% distillate.

### 3.7. Determination of Distillate Colors Using the L*a*b* (CIELab) Method

The method for determining the color (CIELab) of the distillates was conducted in accordance with the protocol described by the International Commission of Claristics [78]. The distillate samples were centrifuged (15 min, 5000 rpm, MPW 350R centrifuge, MPW MED. INSTRUMENTS, Warszawa, Poland) and poured into spectrophotometric cuvettes. The absorption spectra in the range of 380–770 nm were measured using a Konica Minolta CM-5 spectrophotometer (Konica Minolta, Tokyo, Japan).

From the obtained absorption spectra, the CIELab color parameters were calculated: L* (perceptual lightness; ranging from 0 = black to 100 = white), and chromatic coordinates: a* and b*. The a* axis corresponds to the green-red color opposites, with negative values toward green (–100) and positive values toward red (+100), while the b* axis corresponds to the blue-yellow color opposites, with negative values toward blue (–100) and positive values toward yellow (+100). Furthermore, additional color parameters were identified: hue (h_ab_) and chroma (C*_ab_, also referred to as color saturation or intensity). These were calculated using the following formulas [41]:

$$h_{ab}=\tan^{-1}\left(\frac{b^{*}}{a^{*}}\right)$$

$$C_{ab}^{*}={\left[{\left(a^{*}\right)}^{2}+{\left(b^{*}\right)}^{2}\right]}^{\frac{1}{2}}$$

where

h_ab_—hue;

C*_ab_—chroma;

a*, b*—color coordinates.

### 3.8. Analysis of the Volatile Compounds Profile of the Distillate—SPME-GC-MS (Solid-Phase Microextraction–Gas Chromatography–Mass Spectrometry) *[79]*

To analyze the volatile compounds, 0.05 mL of the distillate sample and 2 mL of distilled water were placed in a 10 mL vial containing 1 g of NaCl. A SPME device (Supelco Inc., Bellefonte, PA, USA) coated with 100 μm PDMS fibers and conditioned at 250 °C for 1 h was used for sampling. The device was placed in the headspace and stirred at 300 rpm for 40 min at 40 °C. Next, the SPME device was desorbed in the injector port of the chromatograph system for 3 min. These analyses were performed using a Shimadzu GC-2010 Plus gas chromatograph coupled with a GCMS-QP2020 system (Shimadzu, Kyoto, Japan). The analyzed volatiles were separated using an Rxi^®^-1 ms capillary column (Crossbond 100% dimethyl polysiloxane; 30 m × 0.53 mm × 0.5 μm). The column was heated according to the following program: 35 °C for 4 min, increasing at a rate of 5 °C/min to 110 °C; then increasing at a rate of 20 °C/min to 230 °C; and finally maintaining at a constant temperature for 4 min. Helium was used as the carrier gas at a constant flow rate of 1.0 mL/min. The analyte was injected in splitless mode. Mass spectra were recorded in SEM mode. Compound identification was performed using mass spectral libraries and linear retention indices derived from the C6 to C30 n-alkane series. Semi-quantitative analysis was conducted by measuring the relative peak area of each identified volatile compound using the NIST database.

Cluster analysis (HCA) was performed in Visual Studio Code (version 1.124.2 Microsoft Corporation, Redmond, WA, USA) using Ward’s minimum variance method with Euclidean distance as the similarity measure. The results are presented as a heat map with a dendrogram.

### 3.9. Organoleptic Assessment of Apple Distillate

The organoleptic evaluation method is an in-house development based on PN-ISO 4121:1998 [80]. All organoleptic analyses were conducted by a trained sensory panel consisting of 15 members. Prior to the evaluation, the panelists were required to complete a series of tests to determine their suitability for the role, including assessments of gustatory and olfactory daltonism, as well as individual gustatory and olfactory sensitivity thresholds. The assessors successfully passed the test for distinguishing basic tastes and odors, as well as the taste sensitivity test, in accordance with the PN-ISO 3972:1998 [81] and PN-ISO 5496:1997 [82] standard. Following these tests, they were selected to carry out the organoleptic assessment of distillate samples. The entire sensory panel regularly undergoes validation tests for various food products, including alcoholic beverages. The sensory evaluation was carried out in a dedicated sensory laboratory. Each panelist was seated in a cubicle separated from their colleagues by walls. The temperature within the laboratory was maintained at 25 °C. The beverage samples were served in tasting goblets made of glass, at room temperature, and each sample was marked with a three-digit code.

The basic sensory evaluation of apple distillates was conducted according to a 5-point scale, with 1 indicating a negative evaluation and 5 indicating a positive one. Four quality attributes were evaluated using weighting factors (given in parentheses): color (0.1), clarity (0.1), aroma (0.2), and taste (0.6). The purpose of weighting factors is to increase or decrease the importance of a particular attribute being evaluated. In this case, taste was given the highest weight, as it is the primary sensory attribute of the product under evaluation. The proposed weighting factors comply with the Polish Standard PN-ISO 4121:1998 [80].

Furthermore, the perception of aromas (fruit, herbal, toast, wood, smoke, chocolate, spices, caramel, coconut, vanilla, foreign) and tastes (sweet, sour, bitter, astringent, fruit, herbal, toast, wood, smoke, chocolate, spices, caramel, coconut, vanilla, foreign) were also assessed. A five-point scale was used to assess the perceptibility of specific tastes and odors, with a score of 1 indicating an undetectable taste or odor and a score of 5 indicating an intensely perceptible taste or odor.

### 3.10. Statistical Analysis

The analysis was repeated at least three times, and the results were presented as the arithmetic mean with standard deviation (±SD). The statistical analysis was performed using InStat v. 3.01 (GraphPad Software Inc., San Diego, CA, USA). A one-way analysis of variance (ANOVA) with a post hoc Tukey’s test was used to assess the significance of differences between means. The Kolmogorov–Smirnov test was performed to assess the normality of the distribution.

## 4. Conclusions

The wood species and the thermal treatment of the wood chips imparted specific sensory and chemical properties to the distillates.

Chestnut had the most positive effect on the quality of the distillates of all the wood species. The addition of chestnut wood chips to distillates resulted in the greatest chemical changes and a high rating from the sensory panel. Cherry and oak wood chips had a similar effect. In contrast, distillates made with the addition of juniper wood exhibited the most unusual characteristics compared to other species; they were characterized by the fewest chemical changes relative to the control. This confirmed the first hypothesis and identified medium-toasted chestnut wood chips as the optimal choice for the maturation of apple distillates.

Additionally, it was demonstrated that thermal treatment significantly alters the chemical composition of the wood, affecting the chemical and sensory characteristics of the distillate. These changes varied among different tree species. It is worth noting that chestnut wood, despite its already high content of active compounds, showed a further increase in these compounds when toasted to light and medium degrees. Similar trends were observed in distillates containing oak and juniper wood. Although juniper contains large amounts of essential oils not found in the other wood chips studied, this type of wood exhibited similar behavior when exposed to heat during toasting. Cherry wood chips toasting, however, had a negative impact on the antioxidant properties of the apple distillate, particularly at high toasting levels. The second hypothesis was therefore rejected—cherry and juniper wood chips, regardless of the degree of toasting, are no more suitable for the maturation of apple distillates than chestnut or oak wood.

## Figures and Tables

**Table 1:** Color parameters of apple distillates.

Sample		L*	a*	b*	h_ab_	C*_ab_
Oak	untoasted	94.06 ± 0.22 ^a,c,f^	−1.28 ± 0.15 ^a,d^	8.15 ± 0.86 ^a,d^	171.07 ± 0.23 ^a,f,g^	8.25 ± 0.87 ^a,e^
	lightly toasted	94.07 ± 0.19 ^a,c,f^	−1.30 ± 0.06 ^a,d^	7.97 ± 0.48 ^a,d^	170.70 ± 0.17 ^a,f,g^	8.07 ± 0.48 ^a,e^
	medium toasted	93.69 ± 0.48 ^a,b,c,f^	−1.30 ± 0.24 ^a,d^	8.78 ± 2.13 ^a,e^	171.50 ± 0.44 ^a,f^	8.88 ± 2.14 ^a,d^
	heavy toasted	92.97 ± 1.60 ^a,b,d,f^	−1.60 ± 0.04 ^a^	10.74 ± 1.33 ^a,c,f^	171.43 ± 0.85 ^a,f^	10.86 ± 1.32 ^a,c,f^
Cherry	untoasted	93.12 ± 0.40 ^a,b,c^	−0.65 ± 0.14 ^b,c,e^	12.19 ± 1.79 ^a,b^	176.87 ± 1.08 ^b,c,h^	12.21 ± 1.78 ^a,b^
	lightly toasted	92.12 ± 1.45 ^b,e^	−0.71 ± 0.33 ^b,e^	15.99 ± 4.32 ^b,g^	177.14 ± 1.74 ^b,h^	16.02 ± 4.31 ^b,h^
	medium toasted	92.50 ± 0.30 ^a,b,e^	−0.22 ± 0.22 ^c,g^	14.34 ± 1.33 ^b,c^	179.10 ± 0.96 ^b^	14.34 ± 1.33 ^b,c^
	heavy toasted	93.59 ± 0.16 ^a,b,c^	−1.61 ± 0.07 ^a^	11.49 ± 0.70 ^a,b,f^	172.03 ± 0.15 ^a,d,f^	11.60 ± 0.70 ^a,b,f^
Juniper	untoasted	94.82 ± 0.02 ^c,g^	−0.35 ± 0.08 ^b,c,g^	3.78 ± 0.21 ^d,h^	174.70 ± 0.98 ^c,e,i^	3.79 ± 0.21 ^d,e,i^
	lightly toasted	94.84 ± 0.27 ^c,g^	−0.39 ± 0.11 ^b,c,g^	3.83 ± 1.24 ^d,h^	174.07 ± 0.68 ^d,e,i^	3.85 ± 1.24 ^e^
	medium toasted	94.81 ± 0.21 ^c,g^	−0.60 ± 0.14 ^b,c^	4.31 ± 1.41 ^d,e,h^	172.57 ± 0.81 ^a,e^	4.34 ± 1.42 ^d,e,i^
	heavy toasted	94.54 ± 0.19 ^c,d^	−1.10 ± 0.07 ^d,e,f^	6.09 ± 0.72 ^d,e^	169.70 ± 0.60 ^f,g^	6.19 ± 0.72 ^d,e,g^
Chestnut	untoasted	94.29 ± 0.24 ^c,d^	−1.39 ± 0.20 ^a^	7.17 ± 0.99 ^d,e,f^	169.03 ± 0.06 ^g^	7.30 ± 1.01 ^d,e,f^
	lightly toasted	94.27 ± 0.23 ^c,d^	−1.48 ± 0.28 ^a,f^	7.42 ± 1.32 ^d,e,f^	168.70 ± 0.20 ^g^	7.56 ± 1.35 ^a,e^
	medium toasted	93.66 ± 0.20 ^a,b,c^	−1.71 ± 0.10 ^a^	10.33 ± 0.86 ^a,c,e^	170.57 ± 0.25 ^a,g^	10.47 ± 0.86 ^a,c,f,g^
	heavy toasted	90.91 ± 0.14 ^e^	−1.73 ± 0.02 ^a^	20.01 ± 0.67 ^g^	175.07 ± 0.15 ^h,i^	20.09 ± 0.66 ^h^
Control		95.42 ± 0.12 ^d,f,g^	−0.06 ± 0.01 ^g^	0.57 ± 0.03 ^h^	173.97 ± 1.31 ^d,e,i^	0.58 ± 0.03 ^i^

The same letters within a column indicate no statistical significance at *p* < 0.05 (*n* = 3; mean ± SD).

**Table 2:** Effect of wood species and degree of toasting on the results of the organoleptic evaluation of apple distillates.

Wood Type	Untoasted	Light	Medium	Heavy
Oak	3.65 ± 1.03	3.32 ± 0.72	3.83 ± 0.51	3.21 ± 0.72
Cherry	3.74 ± 0.79	3.74 ± 0.66	3.83 ± 0.54	3.71 ± 0.45
Juniper	3.13 ± 0.56	3.02 ± 0.54	3.08 ± 0.94	3.43 ± 1.03
Chestnut	3.61 ± 0.64	3.58 ± 0.84	3.93 ± 0.67	4.11 ± 0.88
Control	3.11 ± 0.83			

## Data Availability

The original contributions presented in this study are included in the article. Further inquiries can be directed to the corresponding author.
